# Case Report: Dental treatment under general anesthesia and dental management of a child with congenital ichthyosis

**DOI:** 10.3389/fdmed.2024.1481658

**Published:** 2024-10-17

**Authors:** Ryoko Hino, Yuta Chiba, Yuriko Maruya, Manami Tadano, Shinji Otake, Seira Hoshikawa, Yoji Sasahara, Kan Saito

**Affiliations:** ^1^ Division of Pediatric Dentistry, Department of Oral Health and Development Sciences, Tohoku University Graduate School of Dentistry, Sendai, Japan; ^2^ Department of Pediatrics, Tohoku University Graduate School of Medicine, Sendai, Japan

**Keywords:** congenital ichthyosis, pediatric dentistry, enamel hypoplasia, genetic disorder, caries treatment, general anesthesia

## Abstract

Congenital ichthyosis is a disease in which the stratum corneum on the surface of the skin becomes thick from the time of the fetus and the barrier function of the skin is impaired. Congenital ichthyosis is a genetic disorder that causes ectodermal abnormalities and sometimes affects skin, nails, and tooth enamel. Therefore, some patients require special care in their daily life and during dental treatments. Here, the authors report a case of congenital ichthyosis that developed into severe dental caries at two years and nine months of age. The authors performed whole-exome sequencing in his peripheral blood and found that the patient had compound heterozygous mutations in ALOX12B gene (c.159C>G and c.1579G>A), which is responsible for autosomal recessive congenital ichthyosis-2 (MIM#2421000). Mutation of c.159C>G is a nonsense mutation that has never been reported, therefore novel symptoms might have found. The patients exhibited severe caries by hypoplastic teeth. Here, the authors report the treatment of dental caries in a patient with congenital ichthyosis under general anesthesia and its oral management until mixed dentition.

## 1 Introduction

Congenital ichthyosis is a rare genetic skin disorder that exhibits hereditary patterns, such as autosomal recessive, autosomal dominant, X-linked recessive, and X-linked dominant (1). It is caused by abnormal differentiation of epidermal cells and abnormal production, metabolism, and transport of lipids (2). As a result, they affect the barrier function of the skin and the stratum corneum thickens remarkably. Generally, the prognosis of this disorder is good; however, in severe cases, eyelids and lips roll up, and malformation of the auricle is observed, which leads to death in the newborn and infancy (1–3). The children suffering congenital ichthyosis have difficulty in the control of body temperature because of skin dyskeratosis and dyshidrosis and easily develops hyperthermia when the patients cry (2, 4). Therefore, dental treatment for young non-cooperative patients with congenital ichthyosis may have difficulty, and in such case dental treatment under general anesthesia would be one of the treatment plans (5). Congenital ichthyosis includes the following 4 classifications: (i) Keratinolytic ichthyosis including bullous congenital ichthyosiform erythroderma (BCIE), (ii) Harlequin ichthyosis, (iii) Autosomal recessive congenital ichthyosis (ARCI) including nonbullous congenital ichthyosiform erythroderma) and lamellar ichthyosis, and (iv) Ichthyosis syndrome with other organ symptoms (IS). The total number of patients with BCIE was estimated to be 55 (4), and only 220 patients are reported to have been treated for ARCI and IS in Japan (6). Thus, congenital ichthyosis is rare. The genes responsible for this disease are *transglutaminase 1* (TGM1), *arachidonate 12-lipoxygenase, 12R type* (ALOX12B), *cytochrome P450 family 4 subfamily F member 22*, *ATP binding cassette subfamily A member 12* (ABCA12), *arachidonate lipoxygenase 3*, and *NIPA like domain containing 4* (NIPAL4) (7–10). The symptoms vary depending on the type of gene mutation. Recently, systemic symptoms including dermatological and ophthalmological defects have gained attention (5, 11). Although protrusion of the lips, an open mouth, and anomalies of the teeth are observed as oral findings (12), oral and dental evaluations are still not well-investigated. Here, the authors report the case of a 2 years and 6 months-old boy with congenital ichthyosis induced by genetic mutations in ALOX12B, who underwent dental treatment under general anesthesia. The case showed severe dental caries and noncooperative behavior because of his young age and intellectual disability, and the authors conducted dental treatment under general anesthesia and obtained good prognosis.

## 2 Case report

The patient was a Japanese boy with a history of congenital ichthyosis syndrome. He was born at 37 weeks of gestation, and his birth weight and height were 2,562 g and 46.0 cm, respectively. The patient's skin exhibited severe inflammation and scaling. He was diagnosed with suspected Netherton syndrome, and had normal psychomotor development. At 1 year and 7 months of age, he was infected with rotavirus and developed epilepsy, dysphagia, psychomotor developmental delay, cortical blindness, and quadriplegia as aftereffects. In addition, he was allergic to eggs, fish eggs, wheat, soy, and dairy products. Hence, he took four anticonvulsants, four antiallergic agents, a levocarnitine preparation, and a steroid ointment. He had a history of general anesthesia during inguinal hernia surgery at the age of 2 years and cryptorchidism surgery at the age of 2 years and 8 months. After surgery, he developed fever, and discharge was postponed. The patient was one of the fraternal twins. His younger brother was a typical developer, whose congenitally missing teeth were pointed out during a dental health checkup at 2 years and 6 months of age. However, the patient could not undergo a dental health check-up at 2 years and 6 months of age. Instead of undergoing a dental health check-up, he underwent a dental check-up at a dental clinic in a nursing care center, and the dentist pointed out his severe dental caries. At the age of 2 years and 9 months, he was referred to the Pediatric Dental Clinic of Tohoku University Hospital for dental treatment under general anesthesia.

**Figure 1:**
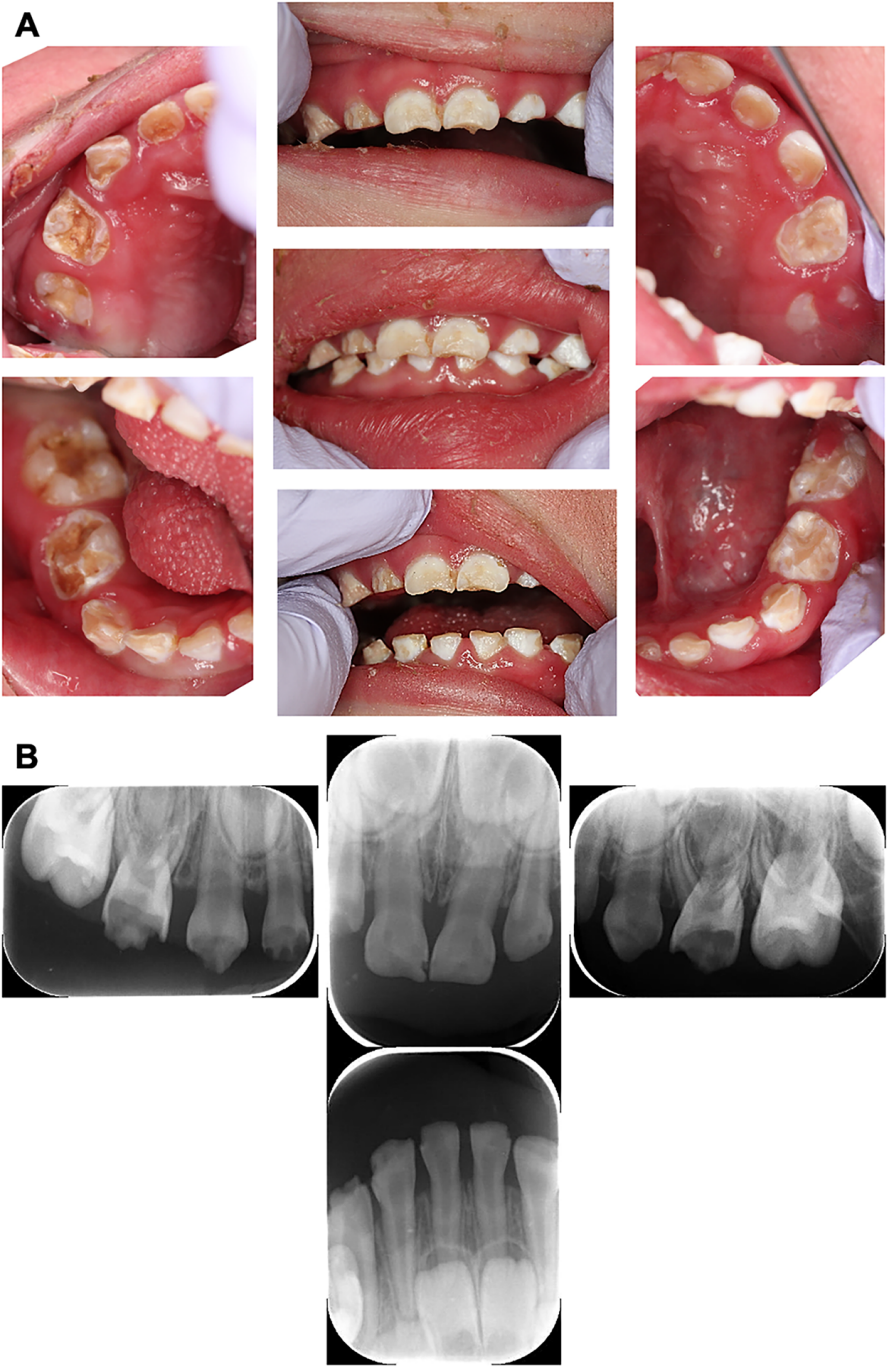
Intraoral and dental x-ray photographs at the initial examination (2 years and 9 months-old). **(A)** Intraoral photographs at the initial dental examination. Teeth showed numerous opaque white and brown discolourations and defects, diagnosed as multiple severe caries. **(B)** Pre-treatment dental x-ray image. The mandibular molars were not able to be photographed because the cooperation status of the patient had deteriorated.

At the first visit, the patient was 78 cm in height (< −3SD) and 9.08 kg in weight (< −3SD). He had flares, scales, dry skin throughout whole body, and a yellow nail on his thumb. The patient was unable to walk independently and used a wheelchair. An oral examination revealed cloudiness and severe caries in the primary teeth. The patient had poor oral hygiene, gingivitis, gingival swelling, xerochilia, and a tongue coat (Figure 1A). In addition, his skin was overstretched and sensitive. He was unable to understand the requirements for dental treatment and declared against dental treatment. The authors discussed this with his parents and decided that his dental treatment would be best provided under general anesthesia. Due to his skin condition, he was at risk of worsening overall status after general anesthesia. Considering this risk, the authors decided to treat him in three settings of general anesthesia for shorter time per treatment. Before starting dental treatment, the patient was checked by a pediatrician, dermatologist, and anesthesiologist to clarify his medical status for general anesthesia. In addition, the authors requested that a pediatrician perform a medical examination before and after general anesthesia.

**Figure 2:**
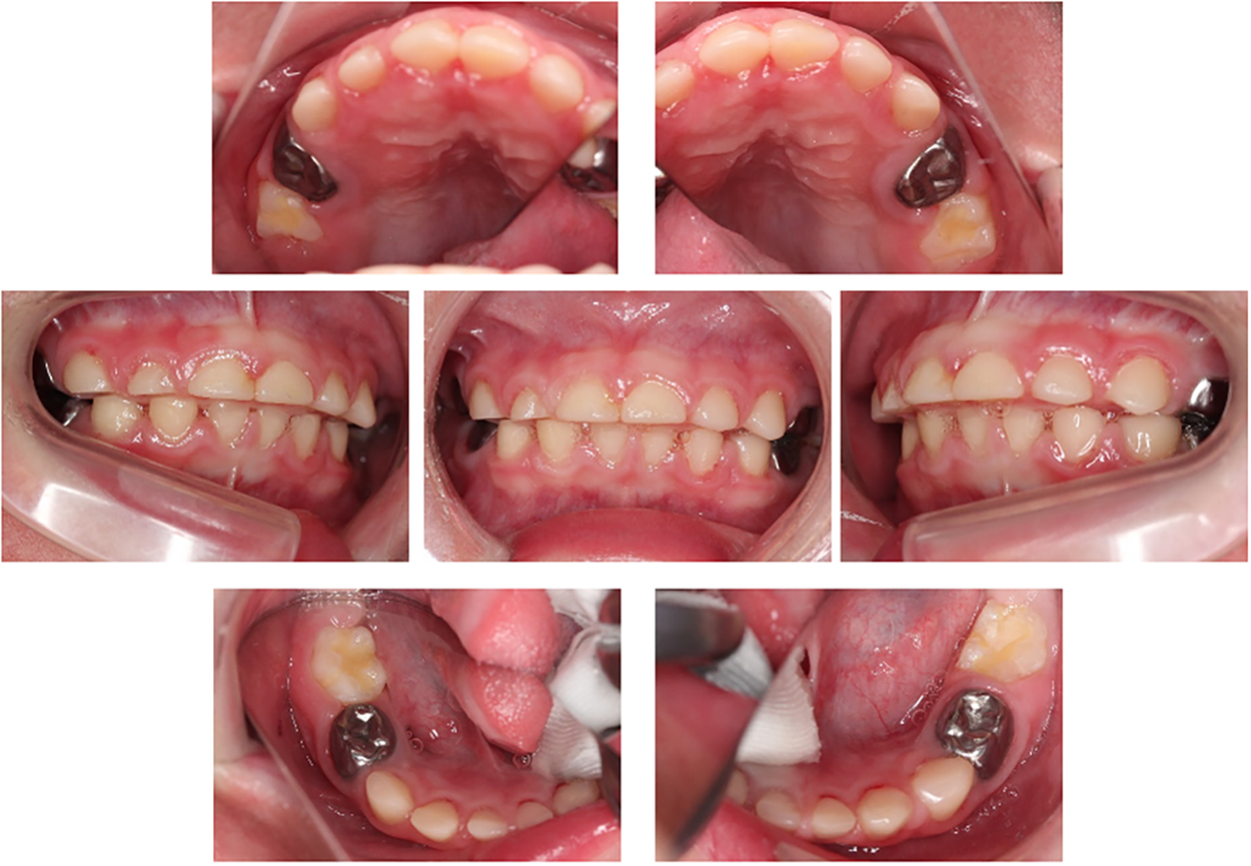
Intraoral photographs at post-treatment (4 years and 7 months old). The patient underwent dental treatment three-times under general anesthesia.

The authors made the diagnosis using dental radiographs (Figure 1B) and divided them into three blocks for treatment. The first treatment was provided when the patient was three years and two months old, and the second treatment was provided six weeks later. At the age of 3 years 5 months, he was admitted to a nursing center for pneumonia, and the third treatment was postponed. Finally, at the age of 3 years and 8 months, the third treatment was administered. Caries of the maxillary right first primary molar, maxillary right primary canine, and maxillary right primary lateral incisor were diagnosed after reaching the pulp. The carious lesion was excavated and the pulp was removed using H-files with frequent irrigation using oxygenated water and 3% sodium hypochlorite. Canals were dried with cotton plugs and obturated with calcium hydroxide preparation (Vitapex®, Neo Dental Chemical Products Co. Ltd.). Strip crowns (3M ESPE) were used for all the anterior teeth, and prefabricated stainless-steel crowns (3M ESPE) were cemented on the upper and lower bilateral first primary molars because the risk of caries was high. The upper and lower bilateral second primary molars had erupted and were restored with a composite resin because their caries were mild. All dental procedures were completed without any complications, and each operation took approximately 2 h. After treatment, the patient recovered uneventfully from the general anesthesia. The final diagnoses and treatment details are listed in Supplementary Table S1. The patient was discharged the day after general examination. Subsequently, the patient is undergoing monthly follow-up for high caries risk and difficulty in oral care. The gingivitis improved with periodic management. In addition, no caries was found, and the oral condition was much better than that before treatment at 4 years and 7 months of age (Figure 2).

**Figure 3:**
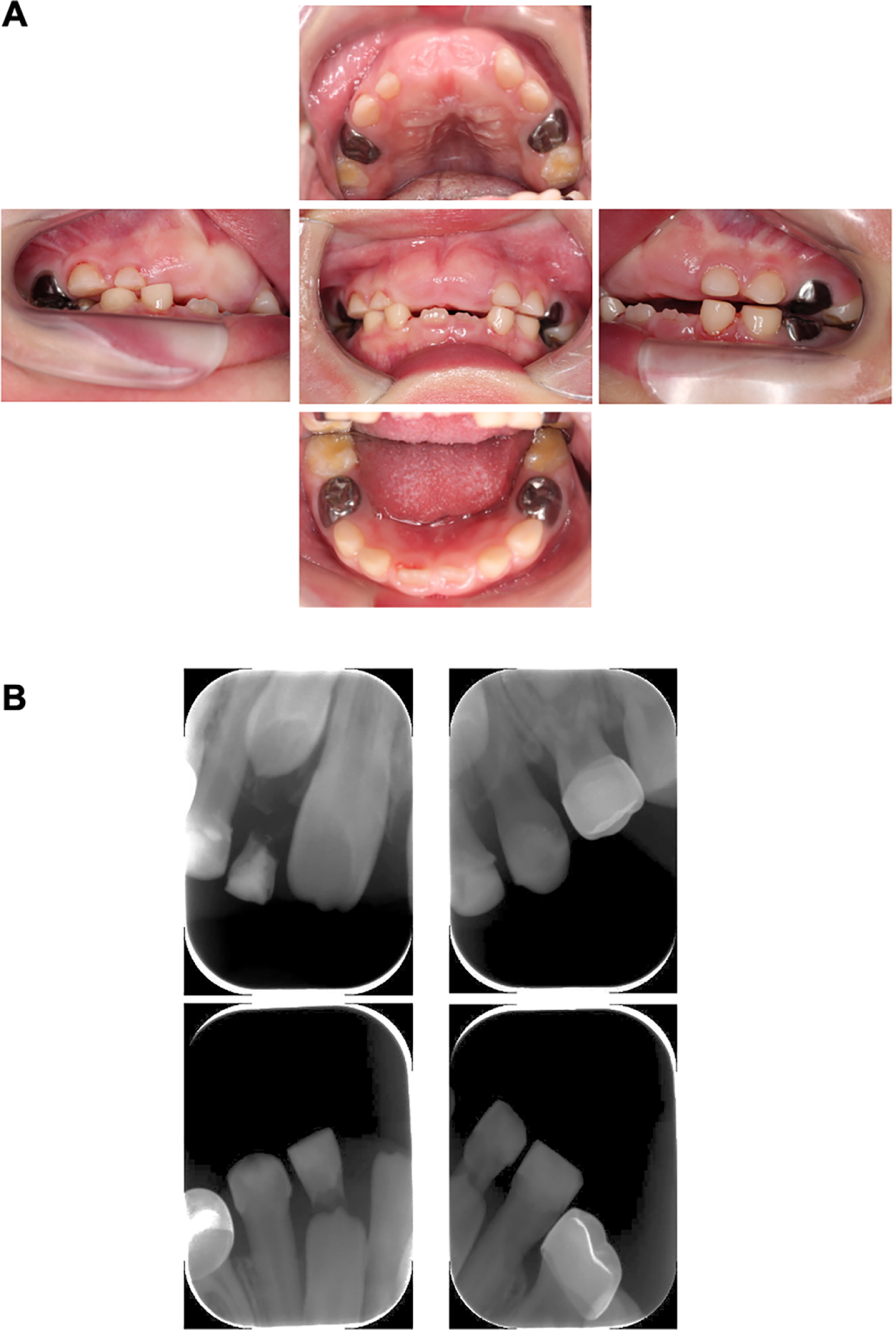
Intraoral and dental x-ray photographs at the time of periodic medical check-ups (6 years and 2 months old). **(A)** Intraoral photographs at the time of periodic medical check-up at 6 years and 2 months-old. The lower central incisors have erupted. **(B)** Dental x-ray image at the time of periodic medical check-up at 6 years and 2 months-old.

**Figure 4:**
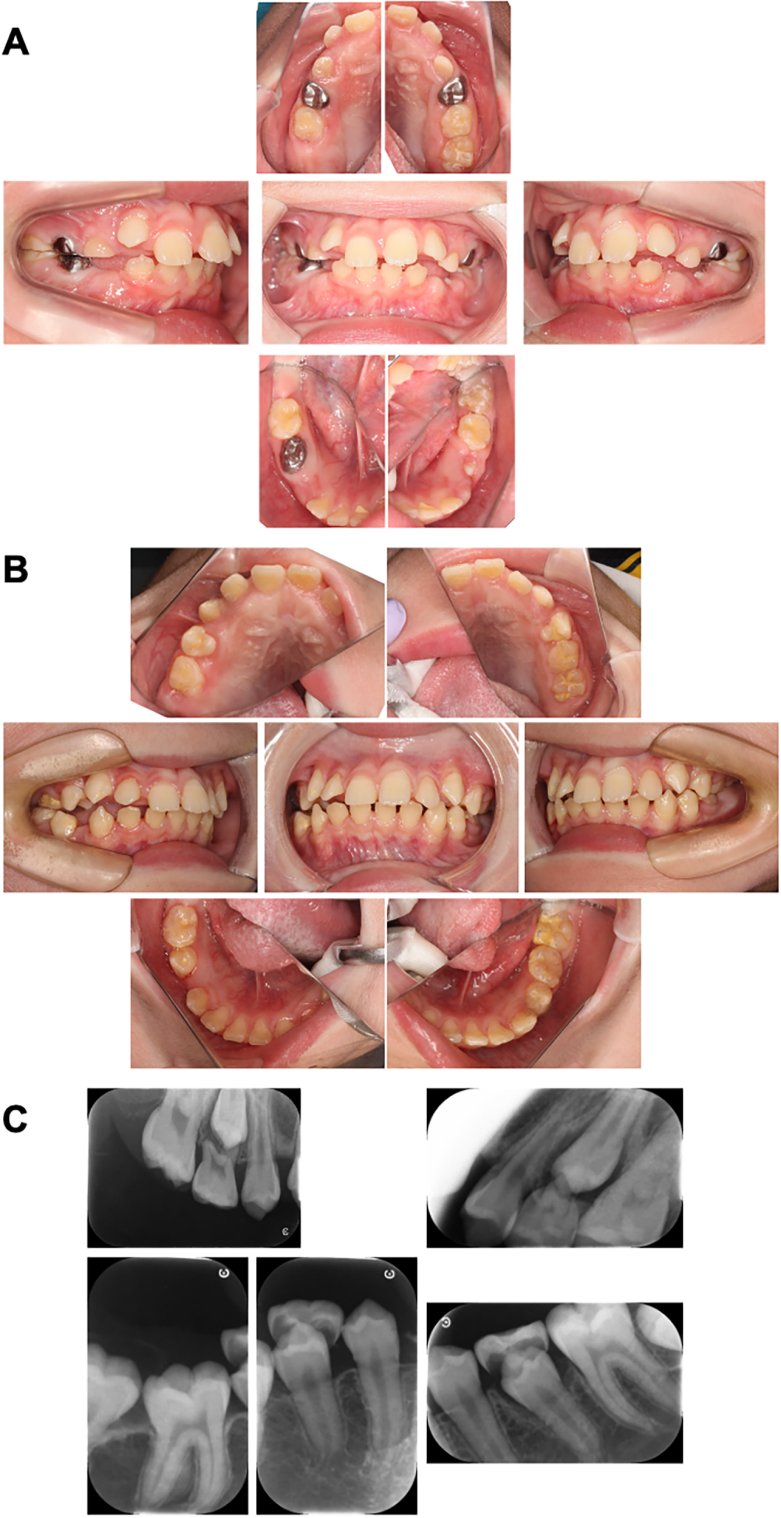
Intraoral photographs at the time of periodic medical check-ups. **(A)** Intraoral photographs at the time of periodic medical check-up at 9 years and 6 months-old. There are no caries and the oral hygiene was improved in this case. **(B)** Intraoral photographs at the time of periodic medical check-up at 11 years and 10 months-old. The primary first molars were replaced with permanent teeth. **(C)** Dental x-ray image at the time of periodic medical check-up at 11 years and 10 months-old.

After the topical application of fluoride using 2% acidulated phosphate fluoride (APF) solution, Fluor Dental Jelly 2%® (Bee Brand Medico Dental Co. Ltd.), the patient's skin around the left corner of the mouth became rough. The authors believe that the cause was hypersalivation after fluoride application and that the acidic solution affected the skin. The authors switched from 2% APF to the neutral type fluoride, Butler fluodent foam N® (Sunstar Inc.). After switching the fluoride application, the patient's skin condition improved. At 4 years 5 months-old, the patient had white and bumpy lesions on his inner cheeks and gums. On suspicion of candidiasis, an antifungal antibiotic syrup was prescribed to the patient by a pediatrician, and the symptoms resolved. The patient had no caries, and the gingival condition was good after teeth treatments. During the dental checkup at 5 years and 10 months of age, the authors found that the lower primary central incisor showed severe mobility because of the exfoliation period of the deciduous teeth. The patient was at risk of aspiration of the exfoliated teeth, which were removed. The permanent central incisors erupted without complications after four months (Figure 3A). At this time, dental x-ray image was taken for examination and there was no delay in the eruption of permanent teeth or dental caries. (Figure 3B) Since then, the authors have continued to maintain his teeth until mixed dentition, and he has no cavities (Figure 4A). Furthermore, pulpectomy-treated teeth was exfoliated as normally and permanent teeth were erupted (Figures 4B,C).

## 3 Discussion

The genes responsible for congenital ichthyosis depend on the type of disease, and recent studies have revealed many etiological genes such as Keratin1 (K1), K2, K10, TGM1, ABCA12, ALOX12B, and NIPAL4 (7, 8, 13). During the early stage of the present case, Netherton syndrome was suspected as a candidate disease. Netherton syndrome is characterized by congenital ichthyosis-like erythroderma, and almost all patients develop atopic dermatitis or asthma as immune disorders (14). The syndrome also presents systemic symptoms such as growth disorders, aminoaciduria, infectivity, poor body temperature regulation, and dehydration (15). Mutations in *serine peptidase inhibitor Kazal type 5* (SPINK5) gene have been identified in genetic diagnosis (16). Since Netherton syndrome was suspected at the age of 2 years and 8 months, the SPINK5 gene mutation was tested by DNA-sequence. However, no mutation of SPINK5 gene was observed in the coding region. Therefore, the gene mutation in this patient was unclear during this period. Owing to recent advances in genetic analysis, whole-exome sequencing has become available to search for genetic mutations in human patients. Whole exome sequencing was performed at 7 years and 1 month of age. Two mutations in ALOX12B gene (c.1579G>A, p.Val527Met and c.159C>G, p.Try53*) were identified as candidates. Previously, only one case of c.1579G>A and p.Val527Met mutations was reported in China (17). However, c.159C>G, p.Try53* mutation has not been reported. This mutation results in a termination codon and stop-gain amino acids in exon 2. Generally, mutations in ALOX12B are not severe but cause congenital ichthyosiform erythroderma and a kink in the ear helix (9, 18–20). The novel mutation (c.159C>G, p.Try53*) is not synthesized after the 53rd amino acid, which may have caused severe conditions such as epilepsy, tetraplegia, and easy infection. Further studies of this mutation are required to clarify the pathogenesis of congenital ichthyosis.

Several genetic disorders in skin show enamel hypoplasia (21, 22). This is because that skin and teeth are both ectodermal derived organs and have similarity in preferentially expressed genes (23–25). While, there are few detailed reports on the oral symptoms of congenital ichthyosis, and the direct effects of the genes responsible for congenital ichthyosis in teeth are unknown. In some cases, teeth develop normally; however, some patients may have defective teeth and are likely to develop caries. In fact, patients with congenital nonbullous ichthyosiform erythroderma present with corneal involvement, hypotrichosis, anhidrosis, nail hyperkeratosis, and dental dysplasia (26). There are other reports of oral and dental findings in persons with ichthyosis, including gingivitis, periodontitis, enamel hypoplasia, delayed primary and secondary eruptions, bruxism, alveolar ridging, bifid teeth, irregular tooth morphology, hyperkeratotic plaques on the tongue, squamous cell carcinoma, mouth breathing, and xerostomia (3, 26, 27). Although the role of ALOX12B during tooth development have not reported yet, the nonsense mutation of this gene might result in severe phenotype and this patient may also have had hypoplastic teeth. Moreover, the patient easily develops hyperthermia because of skin dyskeratosis and dyshidrosis. In particular, in the summer, attention should be paid to an abnormal rise in body temperature, the patient needs to properly hydrate, and adjust the room temperature and clothing. The patient was consuming ionic beverages to prevent dehydration, and the uptake of ionic beverages seemed to have made the patient's caries more severe.

This patient has received medical treatment at multiple hospitals and departments. Before dental treatment under general anesthesia, the authors consulted each doctor about the previous general anesthesia situation, condition, and considerations. The authors confirmed the following points: (i) Regarding epileptic seizure, the patient had several stiffnesses with deviation for a few minutes per day. If the patient experienced an epileptic seizure after general anesthesia, the doctor suggested the use of diazepam or dormicum for treatment. (ii) Regarding tube feeding, before beginning the treatment, the patient was introduced to tube feeding for rehydration 1–2 times a day. If the patient could receive fluids through intravenous drops, the doctor suggested to allow him to take anything orally after surgery. Consequently, the patient was given thickened and soft food because of dysphagia. (iii) Regarding skin condition, the doctor suggested using an ointment and moisturizer until the day before surgery. This prevents skin peeling and blistering by fixing the airway tube with medical tape during surgery. However, the use of tape should be minimized. Additionally, the authors commissioned a pediatrician to visit the patient before and after surgery. The patient was discharged without any complications.

During dental maintenance after surgery, the patient experienced some trouble in the mouth, depending on the immune status of the whole body. The patient's skin around the mouth became rough with 2% APF. A less stimulating fluoride preparation should be used and the excess liquid should be wiped off with a gauze after application. During dental treatment, attention should be paid to the skin surrounding the mouth. The patient was prescribed an antifungal antibiotic syrup on suspicion of oral candidiasis. Originally, the patient had a nail abnormality with a yellow nail only on the thumb. Protracted candidiasis causes the nail color to turn yellow or white, resulting in onycholysis. The patient showed an increase in yellow nails and frequent peeling of the nails after oral candida administration (Supplementary Figure S1). These two symptoms may be related. This patient could not sleep because of laughter seizures at night and had an irregular life rhythm. Furthermore, the patient was often hospitalized because of hypothermia. The authors always considered contacting doctors about changes in his general condition. This disorder requires cooperation from medical specialists and early dental intervention because the symptoms persist throughout life in many cases. In addition, regular oral care and prevention of caries after dental treatment are critical. In this case, there were no caries and the gingival condition was good after teeth treatments. Therefore, proper maintenance should be provided to the patients.

## 4 Conclusion

In this report, the authors present the case of a pediatric patient with congenital ichthyosis who maintained good oral condition after dental treatment under general anesthesia. The case showed noncooperative behavior because of his young age and intellectual disability. For this reason, the case had the risk of hyperthermia during treatment because of congenital ichthyosis and the authors conducted dental treatment under general anesthesia. Medical examination and advise for dental treatment were provided by medical doctor before the treatment and the caries treatments were provided under three times of general anesthesia for shorter time per treatment. Cooperation with other medical specialists, early intervention by dentists, appropriate oral care, and prevention of caries are important for patients with congenital ichthyosis.

## Data Availability

The raw data supporting the conclusions of this article will be made available by the authors, without undue reservation.
