# Supplementary material for: Case Report: Dental treatment under general anesthesia and dental management of a child with congenital ichthyosis
Source: Front Dent Med. 2024 Oct 17;5:1481658. doi: 10.3389/fdmed.2024.1481658 (PMC11797887; doi:10.3389/fdmed.2024.1481658)

Supplementary Material

# Supplementary Tables

**Supplementary Table 1.** The final diagnosis and treatment details performed for the case.

| Final  restoration | Composite resin filling | Preformed crown | Composite resin crown | Composite resin crown | Composite resin crown | Composite resin crown | Composite resin crown | Composite resin crown | Preformed crown | Composite resin filling |  |
| --- | --- | --- | --- | --- | --- | --- | --- | --- | --- | --- | --- |
|  |  |  |  |  |  |  |  |  |  |  |  |
|  |  |  |  |  |  |  |  |  |  |  |  |
| Intermediate  restoration |  | Pulpectomy | Pulpectomy | Pulpectomy |  |  |  |  |  |  |  |
| Diagnosis | C2 | C3, Pulpitis | C3, Pulpitis | C3, Pulpitis | C2 | C2 | C2 | C2 | C2 | C2 |  |
|  |  |  |  |  |  |  |  |  |  |  |  |
| Dental formula | 55 | 54 | 53 | 52 | 51 | 61 | 62 | 63 | 64 | 65 |  |
| Dental formula | 85 | 84 | 83 | 82 | 81 | 71 | 72 | 73 | 74 | 75 |  |
| Diagnosis | C2 | C2 | C2 | C2 | C2 | C2 | C2 | C2 | C2 | C2 |  |
|  |  |  |  |  |  |  |  |  |  |  |  |
| Intermediate  restoration |  | Indirect  pulp capping |  |  |  |  |  |  |  |  |  |
|  |  |  |  |  |  |  |  |  |  |  |  |
| Final  restoration | Composite resin filling | Preformed crown | Composite resin crown | Composite resin crown | Composite resin crown | Composite resin crown | Composite resin crown | Composite resin crown | Preformed crown | Composite resin filling |  |
|  |  |  |  |  |  |  |  |  |  |  |  |

# Supplementary Figures

**Supplementary Figure 1. Photographs of hands and foot at the age of 4 years and 7 months**

The skin became red and was covered with thick keratin. The patient showed an increase in the number of yellow nails and frequent peeling of the nails.


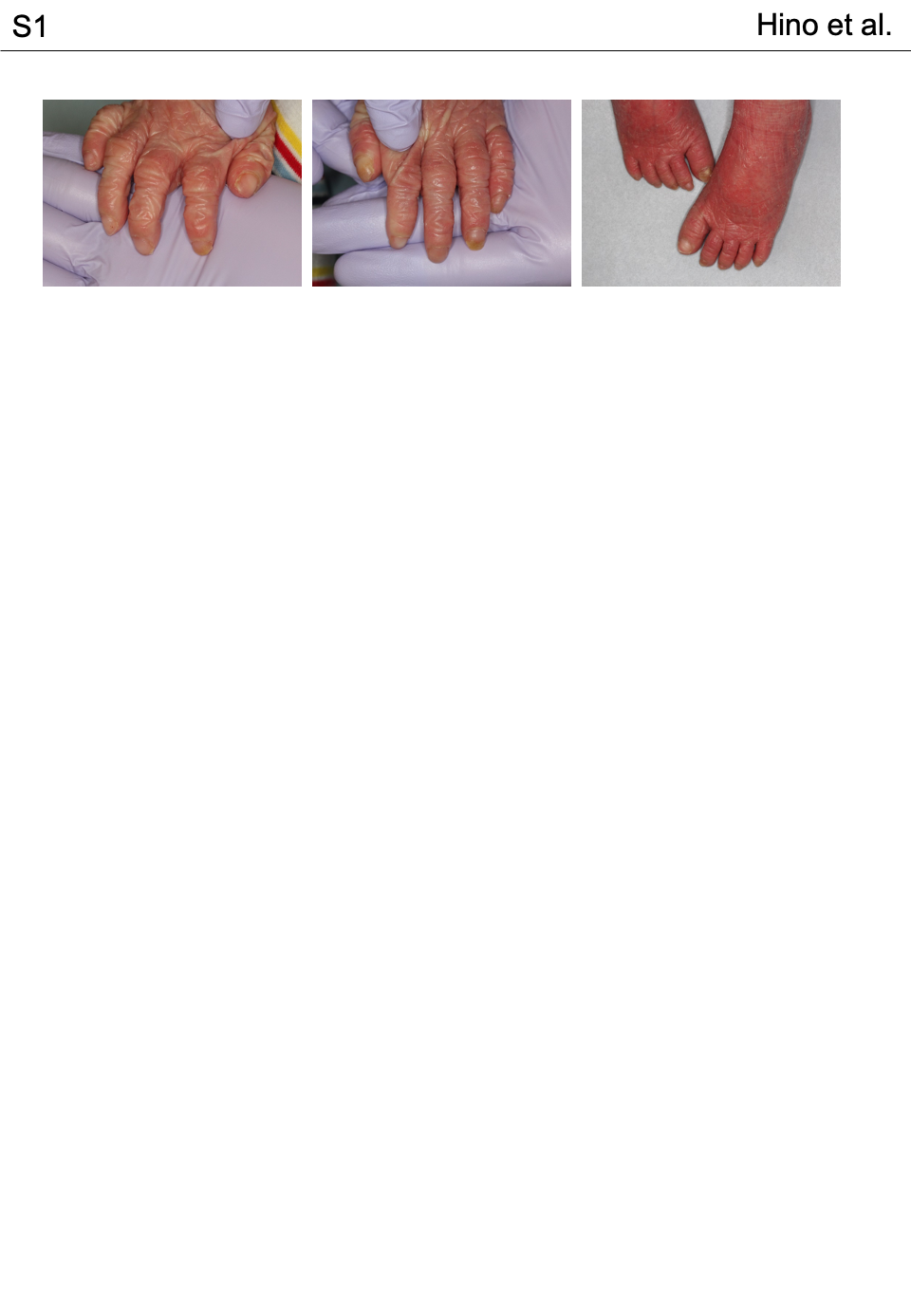

Supplement: Supplementary file 1 [file Table1.docx]
